# Patient Activation: The Cornerstone of Effective Self-Management in Chronic Kidney Disease?

**DOI:** 10.3390/kidneydial2010012

**Published:** 2022-03-03

**Authors:** Courtney J. Lightfoot, Devika Nair, Paul N. Bennett, Alice C. Smith, Anthony D. Griffin, Madeleine Warren, Thomas J. Wilkinson

**Affiliations:** 1 Leicester Kidney Lifestyle Team, Department of Health Sciences, University of Leicester,Leicester LE1 7RH, UK;; 2 Leicester NIHR Biomedical Research Centre, Leicester LE5 4PW, UK; 3 Division of Nephrology and Hypertension, Vanderbilt University Medical Center, Nashville, TN 37232, USA;; 4 Vanderbilt Center for Kidney Disease, Nashville, TN 37232, USA; 5 Clinical & Health Sciences, University of South Australia, Adelaide, SA 5000, Australia;; 6 Leicester Kidney Lifestyle Team, Leicester General Hospital, Leicester LE5 4PW, UK;; 7 NIHR Applied Research Collaboration East Midlands (ARC-EM), Leicester Diabetes Centre, Leicester LE5 4PW, UK;

**Keywords:** patient activation, PAM, self-management, kidney disease, outcomes

## Abstract

The importance of patient activation (i.e., the knowledge, skills, and confidence one has in managing one’s own healthcare) in people with long-term conditions, including kidney disease, is growing. Enabling and empowering patients to take a more active role in their health and healthcare is the focus of person-centred care. Patient activation is recognised as a key construct of self-management, as to effectively self-manage a long-term condition, it is required to enable individuals to actively participate in treatment decisions, prevent complications, and manage risk factors. Identifying an individual’s level of activation can help guide and tailor care, and interventions aimed at increasing patient activation may improve patient engagement and health outcomes. In this review, we explore the concepts of patient activation and self-management, the relationship between patient activation and self-management, interventions aimed at improving these, and what these mean to people living with kidney disease.

## 1. Introduction

The role of self-management is gaining increasing attention in the management of long-term conditions, including chronic kidney disease (CKD). Self-management refers to the means by which people take an active role in their healthcare [1]. In the UK, supported self-management is part of the National Health Service’s (NHS’s) commitment to make personalised care the norm. This emphasises the need to encourage, support and empower people to manage their ongoing health themselves [2]. In order for an individual to be able to look after their health and effectively manage their long-term condition, they need to have the knowledge to understand what to do and why, the skills to be able to perform the required tasks or behaviours, and the confidence that they can do it—this has been termed patient activation [3]. In this perspective, we review our contemporary understanding of patient activation, self-management, and the relationship of these constructs across the spectrum of CKD care. We explore interventions and initiatives that may improve patient activation and self-management behaviours, and we share the personal perspectives of self-management from two people living with kidney disease.

## 2. What Is Self-Management?

**Figure 1.**
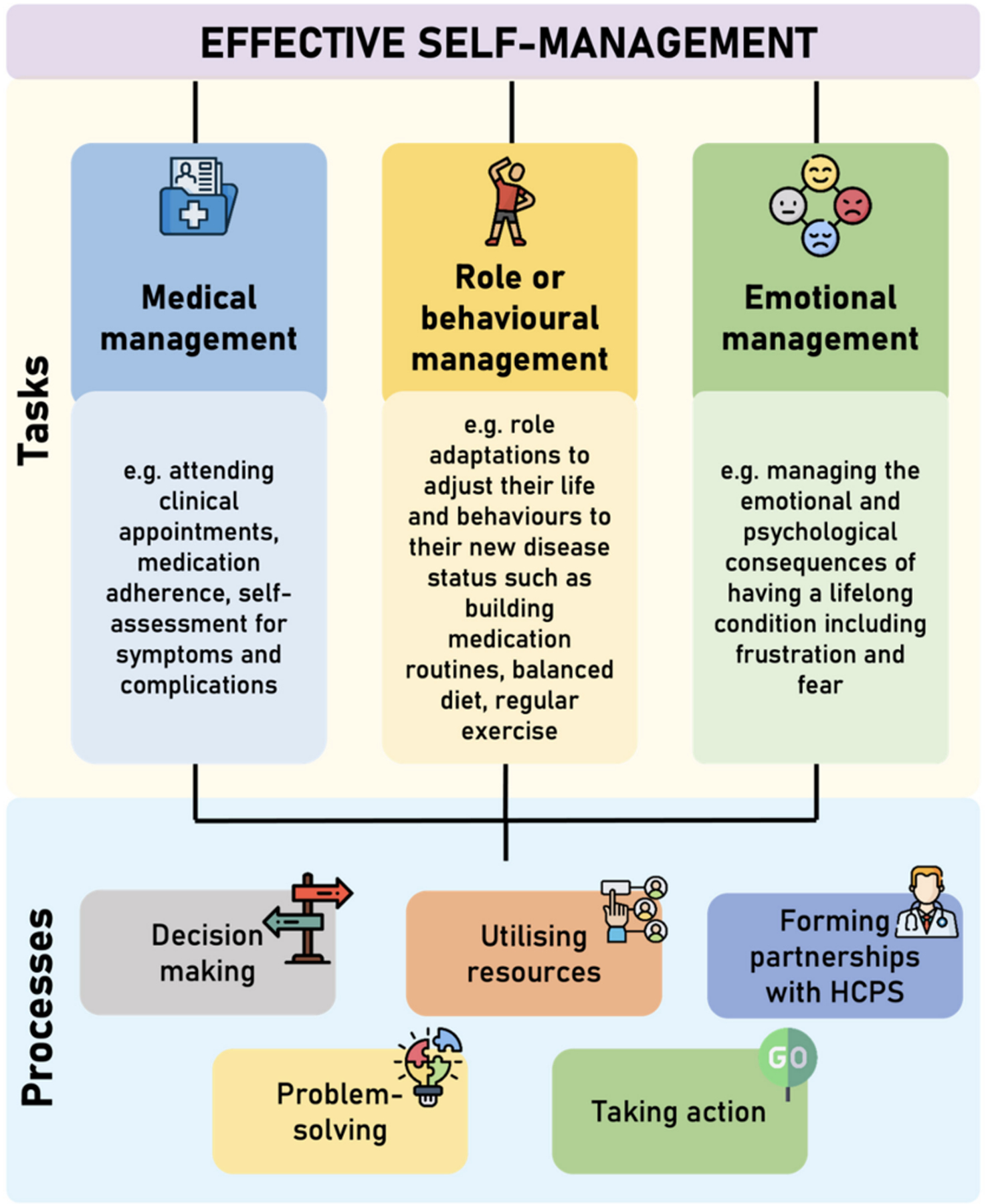
Core tasks and processes involved in effective self-management (adapted from Corbin and Strauss [7] and Lorig and Hoffman [6]. HCPs = Healthcare professionals.

Self-management is an essential component in chronic disease management to empower patients to take a more active role their health [4]. There is no universally accepted definition of self-management; however, many definitions include the maintenance of wellness and the management of chronic conditions [5], whereby an individual takes responsibility for all, or some, aspects of the day-to-day management of their condition [6]. Originally identified by Corbin and Strauss [7], effective self-management comprises of three core tasks (medical management, role or behavioural management, and emotional management), which are underpinned by five key processes (decision making, utilising resources, forming partnerships with healthcare professionals, problem solving, and taking action) [6]; this is displayed in Figure 1. Self-efficacy is required to execute and engage with this complex set of tasks and processes.

The prioritisation of self-management is becoming increasingly prevalent in clinical guidelines for long-term conditions. The NHS Long Term Plan incorporates supported self-management within personalised care to encourage, support, and empower people to manage their ongoing physical and mental health conditions themselves to live well with their condition [2]. This includes the provision of self-management education to help people with long-term conditions to develop the knowledge, skills, and confidence they need to manage their own health care effectively [2].

### Self-Management and CKD

The promotion of self-management behaviours is increasingly being considered as a standard of care in the effort to decelerate CKD progression and prevent complications in CKD management guidelines. Kidney Disease: Improving Global Outcomes (KDIGO) clinical practice guidelines include self-management as a component of CKD models of care, and recommend information, advice, and education to support self-management behaviours should be incorporated into the treatment plan at all stages of CKD [8]. The UK National Institute of Care and Excellence (NICE) guidelines for CKD assessment and management recommend that systems are in place to support self-management and enable people with CKD to make informed choices [9]; one method is to provide people access to their medical data through information systems, such as Renal Patient View/Patient Knows Best (secure online record of own health and care information, linked to medical records), to encourage and help them self-manage their CKD [9].

The goal of self-management education is to identify strategies that can be used to help patients manage their condition(s) whilst leading full, active, and productive lives. For those living with CKD, self-management behaviours range from medication adherence, health monitoring, and symptom monitoring to lifestyle modifications (e.g., increasing physical activity and eating an appropriate diet), which reduce cardiovascular, CKD progression and general health risk factors [10]. Learning to live with and coping with the emotional consequences associated with CKD are important in looking after one’s mental health [11] and can facilitate an optimistic view and positive attitude towards their condition, health, and life [12]. Engaging in self-management behaviours can reduce symptom burden, improve quality of life, and potentially slow the progression of CKD [13,14]. To help people with CKD self-manage, it is widely recommended that individuals are aware of their diagnosis, involved in shared treatment decisions, provided access to their medical data, and given information on blood pressure control, exercise, diet, medication management and smoking cessation [9].

## 3. What Is Patient Activation?

Patient activation is a dynamic behavioural concept that describes the degree to which individuals understand their role in their healthcare and how competent they feel in performing that role [3]. The term patient activation refers to both a state and a process [15] and is defined as the knowledge, skills, and confidence an individual has in managing their own health and healthcare [16]. Patients can move from states of low activation (disengagement and overwhelmed) through to high activation (maintaining behaviours) [16,17]. There are four activation levels on this continuum (Table 1).

### 3.1. Theories of Patient Activation

Patient activation is modifiable and incorporates elements of self-efficacy and readiness to change [3,18]. Consequently, patient activation is related to a number of other concepts [3]. Like the stages of change in the Transtheoretical Model (TTM) [19], activation is changeable in both directions and individuals can move both backwards and forwards through the levels. As such, whilst activation can improve, particularly with appropriate support, it is also possible for activation to decline [20]. Declines in patient activation may result from acute health events, hospitalisation, a worsening of health status, and perceived lack of energy and/or time to engage in illness-related activities [21].

As individuals progress to higher levels, they are more likely to engage in and make improvement in health promoting behaviours that promote successful self-management and prevention of poor health [3,22]. However, unlike stages of change that assess how likely an individual is to adopt a desired health behaviour, patient activation considers the individual’s self-assessment of their behaviour-specific skill level, their belief about the importance of the health behaviour, their confidence in adopting the desired behaviour, and their ability to maintain the behaviour in times of stress. The concept of patient activation more closely aligns the ‘Integrated Theory of Health Behaviour Change’ (ITHBC) and its constructs, including condition-specific knowledge/beliefs, self-regulation of skills and abilities, and active engagement in self-management behaviours [23].

Patient activation is moderately correlated to health literacy; however, the two concepts differ. Health literacy is a predominantly a skills-based construct and does not include motivational elements, and so a person may gain the requisite skill set but not the mindset to take action [24]; thus, using health literacy alone to determine the needs of an individual may miss opportunities to activate them [3]. Whilst similar to a number of concepts, patient activation is a better predictor of healthy behaviour over a wider range of outcomes such as health information usage, active provider choice, healthcare appointment preparation quality, and health-related and treatment-related decision-making [25–28].

### 3.2. Patient Activation and Health Outcomes

In long-term conditions, patient activation is associated with a variety of clinical indicators, health outcomes and health behaviour, and is a significant predictor of health service utilisation, healthcare costs, and patient experience [1,3,18,29,30]; higher levels of patient activation are associated with improved self-care activities (i.e., activities undertaken to manage general health and well-being, such as eating healthy foods and physical activity), self-management behaviours (i.e., behaviours central to managing long-term condition, such as adhering to medication regime), health outcomes, adherence to recommended health practices and interventions, improved quality of life, fewer hospitalisations, and lower healthcare costs in people with long-term conditions [1,31–34]. In the UK, people with long-term conditions who had the highest level of activation experienced 32% fewer attendances to emergency departments (ED) and 38% fewer emergency admissions than those with the lowest level of activation [35].

Many individuals (43–60%) living with CKD display low levels of activation [36,37], and often lack knowledge and understanding about the need for self-management and how to perform the necessary behaviours [38]. Those with low activation are more likely to be older, have lower kidney function, have a greater number of comorbidities, lower haemoglobin values, and a great number of cardiovascular disease risk factors [37]. Lower levels of patient activation are strongly associated with lower levels health literacy and higher levels of depression and anxiety [39]; conversely, higher levels of patient activation are strongly associated with higher health-related quality of life and reduced symptom burden [40]. To date, there is a lack of evidence that increasing patient activation is longitudinally associated with better CKD-specific clinical outcomes (e.g., CKD progression); however, ongoing studies will address this.

## 4. Patient Activation and Its Role in Effective Self-Management

As discussed previously, effective self-management involves a multifaceted set of tasks and processes. It is now becoming evident that having the appropriate knowledge, skills, and confidence (i.e., patient activation) and the ability to utilise these to manage their disease, identify and access resources and support may be a fundamental component of effective self-management behaviour [11,41]. Patient activation for self-management is only the first step in the process about how best to meet the needs of self-management [42]. Activating individuals to be a participant in their own health and engage in self-management activities is of critical importance in improving overall health and health-related quality of life [43].

Knowledge about a long-term condition and its treatment is an important component of self-management and patient activation [17]. Having the necessary skills and knowledge of one’s own condition will result in better levels of activation [44], and increased activation is followed by improvements in self-management behaviours [18,45]. However, differing levels of self-management can be influenced by levels of activation. Accompanying symptoms can adversely influence patient activation impacting on daily living and self-management activities [46]. In addition, a high burden from disease and treatments may challenge patient activation and a patient’s ability to self-manage [39], which can be further complicated by a high prevalence of depression and anxiety [47]. Given the individual symptoms and outcomes in each condition, the use of patient activation to promote successful movement across levels of activation is key [43].

Increasing patient activation aims to facilitate behaviour change and improve health outcomes. Positive changes in activation are related to positive changes in a variety of self-management skills in individuals with long-term conditions, such as engaging in regular exercise, managing stress, paying attention to diet, and taking medications [48]. In addition, activation levels have been shown to be correlated with disease specific behaviours; highly activated individuals with diabetes are more likely to take medication as directed, read food labels, and read potential side effects when prescribed a new medication [48]. Similar findings have also been reported in other conditions such as cardiovascular disease [49]. To our knowledge, the relationship between patient activation and CKD-specific behaviours has not yet been explored; however, this has previously been identified as an area for future research [50].

## 5. Measuring Patient Activation and Self-Management

There are a number of measures that assess self-management and patient activation (summarised in Table 2), these include the Chronic Kidney Disease—Self-Management Knowledge Tool (CKD-SMKT) and Patient Assessment of Care (PACIC). However, it is the Patient Activation Measure (PAM) that has become the most used instrument to assess patient activation.

Currently, the PAM-13 is the only validated, evidence-based tailoring tool to support services in building an individual’s skill, knowledge, and confidence to manage their health and care. The PAM-13 has been shown to be a reliable and suitable measure to assess patient activation in people with long-term conditions, including kidney disease [50]. Given the complex relationship between patient activation and self-management, concerns have been raised about the PAM-13’s predictive ability [39], as the PAM-13 assesses an individual’s perceived ability to engage in self-management and not their actual ability [50]. Despite this, the PAM has been shown to be effective as a method to quantify the patient’s ability to conduct self-management [53].

## 6. Strategies to Implement the Patient Activation Measure (PAM)

The PAM-13 may a useful screening tool to tailor education, a quality indicator for delivery of care, or as an outcome measure [54]. It is currently used in three ways: (a) to inform clinical appointment discussions with the patient, (b) incorporated into electronic health records and used to structure patient care, and (c) for research and evaluation.

### (a) Tailoring education and healthcare discussions

The PAM-13 can help clinicians anticipate the type of discussion and guidance a patient may benefit from before an appointment begins, and the questions associated with the PAM provide a structure for conversations about health behaviours [3]. For example, when an individual with CKD comes in for an appointment, a clinician could use the activation questions as a guide to explore how they are coping and managing their condition, and how they feel about making some lifestyle changes to reduce their health risks (i.e., cardiovascular disease risk, risk of CKD complications etc.). If the patient feels confident and motivated, then the clinician could provide more detailed information or may help them develop a structured diet and exercise plan [15]. If the patient feels overwhelmed or powerless, then the clinician may spend time to understand why the patient feels this way and help them determine some manageable tasks that can reduce their risk level while building their confidence [15].

A recent review examining the use of PAM-13 to tailor care for patients with long-term conditions identified a number of enablers and barriers in the development and implementation of tailored interventions based on levels of patient activation [55]. An improved understanding about the purpose and value of using the PAM to tailor interventions, alongside well-defined administration processes that allow for the flexibility required to appropriately inform patient care, are two key factors that clinicians should consider to enable the implementation of the PAM-tailored interventions in clinical settings [55]. Delivering care that is designed to activate patients, including interventions and motivational interviewing, not only increases patient activation but can have greater benefits for the patient improving their health status and quality of life [56,57].

### (b) Assessing quality of care

The concept of patient activation, and its measurement as an indicator of quality of care and effectiveness, is receiving increasing attention from healthcare services across the world. The PAM can be incorporated into electronic records and used to structure patient care.

In the UK, the PAM was piloted in the NHS, through the UK Renal Registry (a renal database containing clinical information), as an outcome measure as part of the ‘Valuing Individuals: Transforming Participation in Chronic Kidney Disease’ work programme [36]. The PAM questionnaire is currently being used by the UK Renal Registry (renalreg.org/) and collected as part of the patient-reported outcome measures (PROM) survey (‘Your Health Survey’) [36,58] and is recommended by NHS England [59], and is available through the Health Systems Support Framework [59].

In the United States, the PAM-13 is used as a quality metric by the Centres for Medicare and Medicaid Services for value-based care models under the Advancing American Kidney Health Initiative, and is included in two options of the Kidney Care Choices payment model of the Centres for Medicare and Medicaid Services (the Kidney Care First and the Comprehensive Kidney Care Contracting models) [60]. These models aim to improve the cost and quality of care along the entirety of a patient’s kidney disease care continuum from non-dialysis through dialysis, transplantation, and the end of life through the implementation of key quality metrics. Nephrology practices participating in these models will receive capitated payments based on PAM-13 scores. The PAM-13 has also been endorsed by the National Quality Forum Quality Positioning System and included in a framework of patient-reported outcomes from the Kidney Care Quality Alliance, an organisation dedicated to developing dialysis-facility performance measures for use in end-stage kidney disease quality programs in the United States.

### (c) Using PAM as an outcome in research and evaluation

The PAM can be utilised in research and evaluation as an outcome measure to assess patient activation and changes of scores over time. In particular, the PAM can be used as an outcome measure for interventions designed to improve health outcomes, or increasing self-management/self-efficacy behaviour [61]. Whilst the evidence to support the use of PAM is well-established in some long-term conditions, such as diabetes, additional evidence is needed to determine the association of patient activation and clinically meaningful outcomes in kidney disease [60].

Few studies have used the PAM in CKD, with many reporting cross-sectional associations between patient activation and clinical characteristics. Nair and Cavanaugh [60] have described the majority of these studies in detail, including information on study design, the kidney disease subpopulation tested, the prevalence of high and low activation levels in each study, and outcomes associated with these activation levels. In summary, low activation is associated with being older [34,36,37,62], having CKD [63], lower eGFR [37,64], haemodialysis vs. transplant and earlier stages of CKD [36,37,40,62,64], higher decisional conflict about treatment options with lower CKD related treatment satisfaction [65], lower medication adherence [66], higher symptom burden [36,40], greater number of comorbidities [37], and poorer quality of life [34,36,40,62]. Wilkinson et al. [37] found that people with low activation had a 17% greater number of cardiovascular disease risk factors, which included being older and having diabetes. No significant associations between activation scores and eGFR decline [64], hospitalizations or emergency department visits [67], glycaemic control and blood pressure [68] have been identified.

## 7. Considerations of Using the PAM

Using PAM for tailoring care is more complex and less understood than using it as an outcome measure, this can be partly attributed to the broad and inclusive nature of PAM (i.e., it is not disease-specific and can be used across different patient groups) [69]. This may limit the relevance and usefulness of the results as the they are not specific enough to patients’ needs [54,70]. The PAM does not ask whether one actually engages in successful self-management or preventive behaviours [16]. Consequently, patients can be fully activated without optimising their health, and activation does not demand that healthful behaviour be pursued above other interests that promote the patient’s wellbeing [15]. Several hypotheses have been identified that suggest different and complex relationships between PAM scores and outcomes, including tailoring care based on the PAM improves efficiency and outcomes, the PAM can be seen as an outcome in itself alongside other outcomes, and use of the PAM in itself improves outcomes through promoting patient-centredness and involvement [61].

Whilst the PAM has a wide range of potential uses and functionality in the context of person-centred care, it is imperative that the application of PAM is appropriate, and the use of PAM generated data is well-defined [71]. Using PAM in clinical settings to tailor care may demand more flexibility in the administration approach compared to when used as an outcome measure for an intervention [55]. As PAM is incorporated into a meaningful metric in kidney care and service, more information is required to understand its utility, and its association to health outcomes in those with CKD [39]. Another challenge with using the PAM-13 is the licensing fee; however, the fee is intentionally low to encourage adoption [3]. Currently, in the UK, the licence cost associated with the PAM-13 is funded by NHS England and NHS Improvement as part of a national agreement.

## 8. Increasing Patient Activation and Self-Management in CKD

**Figure 2.**
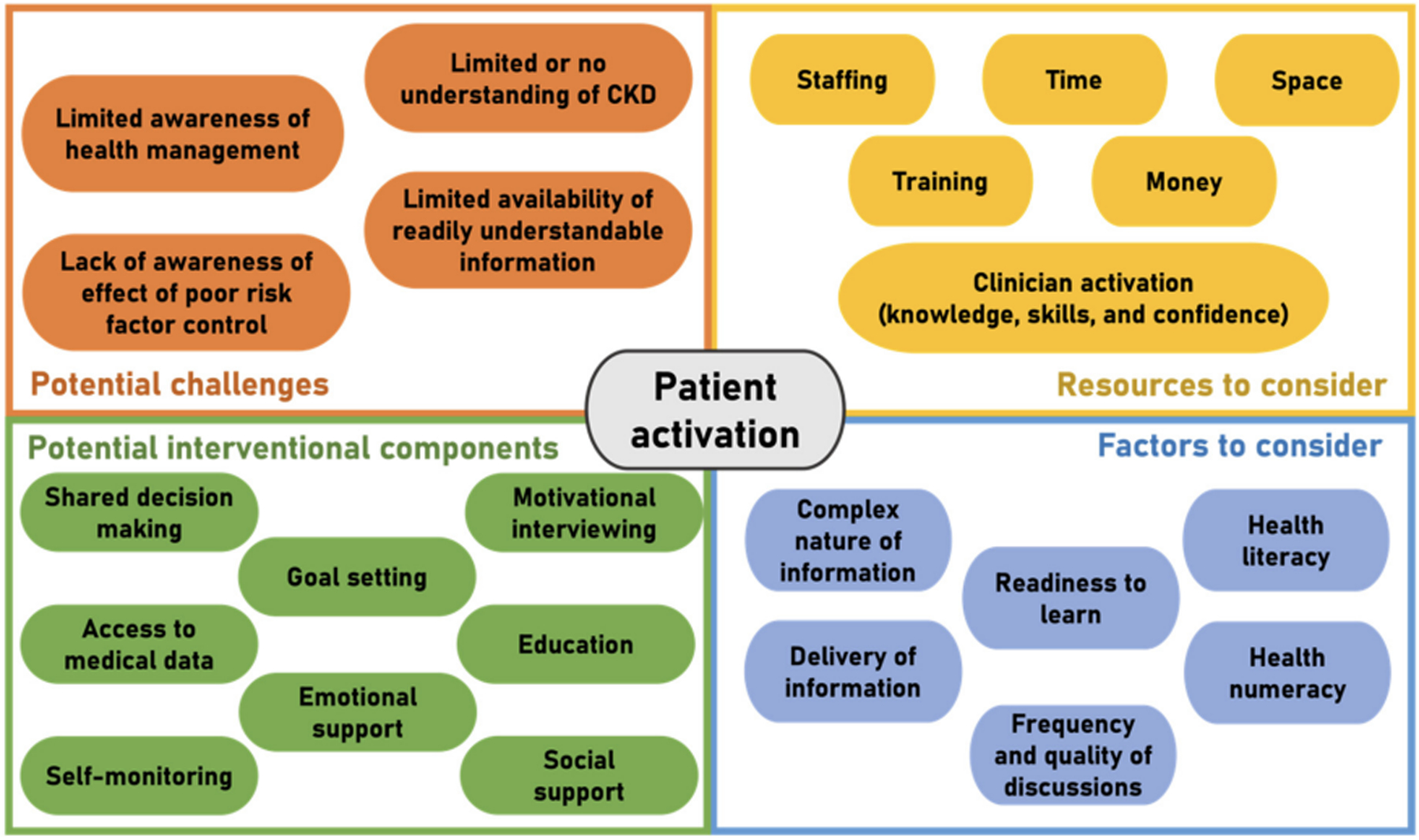
Overview of potential challenges, factors, and components to consider when developing interventions designed to increase patient activation in CKD.

Studies designed to increase self-management behaviour in CKD have often targeted and measured discrete self-management components or clinical factors (e.g., disease-specific knowledge, self-management skills, and/or self-efficacy) [72]. When self-management has been measured, the self-report instruments used vary and, to our knowledge, there are no published interventions designed to specifically target patient activation, particularly assessed using the PAM-13, in those living with CKD. Integration of CKD patient activation may require changes to staff training, delivery of patient education, promotion of interventions, changes in care delivery, resource allocation, and billing processes [73]. An overview of potential challenges, factors, and interventional components to consider when developing interventions designed to increase patient activation, and subsequently self-management, in CKD is displayed in Figure 2.

Indeed, despite evidence suggesting patient activation has important associations with health outcomes, even in other long-term conditions, interventions aiming to improve patient activation are scarce although not non-existent. For example, a study by Deen et al. [74] on community health centres in the USA found that an intervention focusing on building question formulation skills delivered to 252 patients prior to their physician visit significantly increased overall the PAM-13 scores by between 7 and 10 points. A third of participants also moved from lower levels of activation to higher levels post intervention. Those who specified they preferred a more passive role showed greater increases in PAM-13 scores than those who preferred a more active role. In addition, in 233 individuals from Australia living with type 2 diabetes, their PAM-13 score increased by 10 points following attendance at the Diabetes Education and Self-Management for Ongoing and Newly Diagnosed (DESMOND) program—a structured diabetes self-management education. Of the participants exhibiting an increase in patient activation, 87% experienced a clinically significant (>5 point) increase, with an 86% reduction in the proportion of participants scoring in the lowest PAM level [75]. Home-based educational interventions have also shown favourable effects in participants with type 2 diabetes [76]. In a 12-week randomised control trial (RCT) of mainly hypertensive patients in the USA, Solomon et al. [30] showed a patient portal featuring interactive health applications accessible via the Internet was able to increase PAM-13 scores by 10 in those with levels 1–3 activation (although by just 2 points in those in level 4).

A common, yet important, finding across most trials using the PAM-13 as an outcome is that patients in the lowest activation levels experience the greatest increases in PAM-13 post-intervention, whilst those in the highest levels do not experience much improvement [30,75,76]. This may be partly attributed to the ceiling effect that exists in the PAM-13 (e.g., Lightfoot et al. [50]) and is an important consideration in the design of interventional trials.

## 9. Patient Perspectives on Improving Patient Activation to Improve CKD Self-Management

Patient activation is not only linked to clinical and economical outcomes, but also to the patients’ own experiences. Those who are more highly activated have significantly more positive experiences, including higher-quality interpersonal exchanges with doctors, fewer care co-ordination problems, and better care with more out-of-office contact [17,18,52,77–79]. It is suggested that highly activated patients have the skills and confidence to shape more productive interactions with their clinicians, and are more adept at getting their health care providers to be responsive to their needs [3]. In Boxs 1 and 2, we present two patient perspectives on the role of patient activation and self-management.

## 10. Conclusions

Interest in patient activation is growing, and it is increasingly being adopted in the delivery of person-centred care and the management of long-term conditions. In this review, we present an overview of patient activation, self-management, and the relationship between the two concepts. We have described how to measure patient activation and summarise interventions aimed at increasing patient activation in people with CKD. Activating patients can help empower them to take a more active role in looking after their health and managing their kidney disease. Currently the PAM-13 is the most appropriate instrument to measure patient activation in people with CKD. However, no interventions aimed at increasing PAM-13 in people with kidney disease presently exists. Patient activation has great potential for use by both clinicians and researchers in delivering tailored care and interventions to improve the health and quality of life of people living with kidney disease. Can patient activation be considered as the cornerstone of effective self-management in CKD? Patient activation encompasses all the necessary ingredients for effective self-management (i.e., knowledge, skills, and confidence). However, more evidence from CKD-specific populations is needed to show how changing patient activation can positively affect self-management behaviour.

## Figures and Tables

**Table 1.** Description of the four patient activation levels of the Patient Activation Measure.

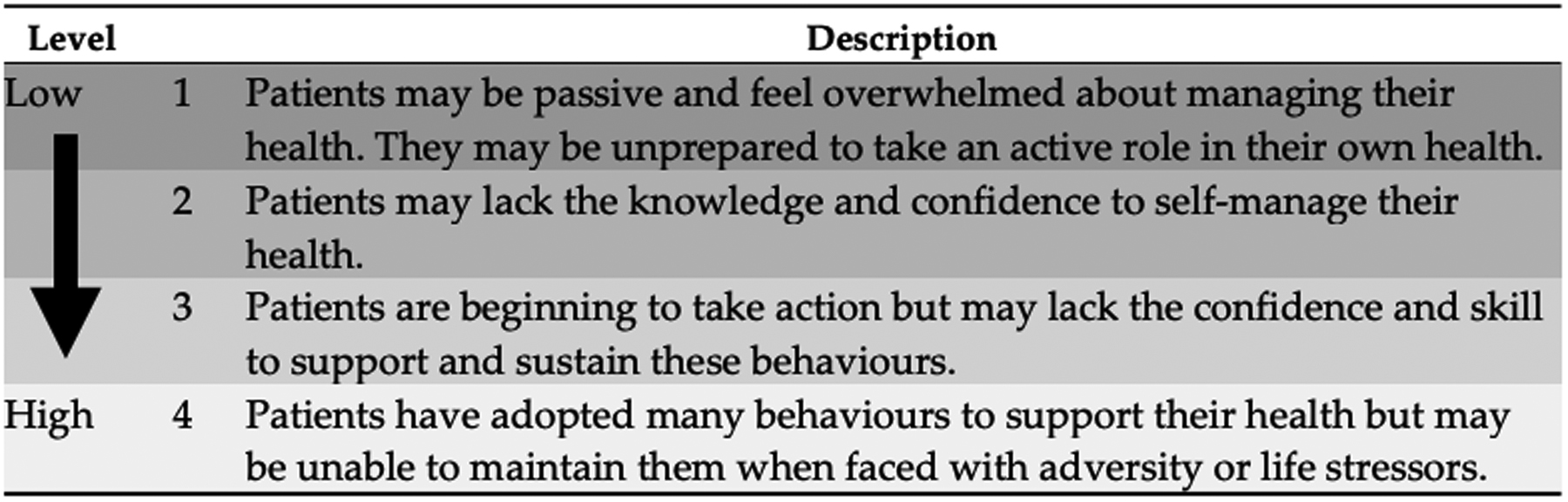

**Table 2.** Commonly used tools or measures that assess self-management and patient activation.

Measure	Description
Chronic Kidney Disease—Self-Management Knowledge Tool (CKD-SMKT) [51]	The CKD-SMKT is a validated 11-item questionnaire, which comprises of several statements of self-management behaviours to which the respondent must indicate if they believe this is ‘true’, ‘false’ or ‘I do not know’, and if they have done this in the last six months (yes or no). Respondents are asked how much they know about their kidney health and to rate this on a five-point Likert scale from “I know everything I need to know” to “I know nothing”. The CKD-SMKT assess CKD disease-specific knowledge of self-management, which is associated with higher patient activation and improved self-management behaviours.
Patient Assessment of Care (PACIC) [52]	The PACIC is a validated 20-item tool to assess the extent to which patients with a long-term condition receive care that aligns with the Chronic Care Model (CCM). The items are aggregated to form five subscales: (1) patient activation, (2) delivery system design/decision support, (3) goal setting/tailoring, (4) problem solving/contextual, and (5) follow-up/coordination. Whilst these subscales are congruent to the components of CCM, they do not perfectly map onto the CCM components. Respondents rate how often they experienced the content described in each item during the past six months. Each item is scored on a five-point Likert scale from “almost never to “almost always”. Patient activation (i.e., actions that solicit patient input and involvement in decision-making), goal setting (i.e., acquiring information for and setting of specific, collaborative goal), and problem-solving/contextual (i.e., considering potential barriers and the patient’s social and cultural environment in making treatment plans) counselling all map onto self-management support in the CCM.
Patient Activation Measure (PAM) [16]	The PAM-13 is the short form of the 22-item PAM [17] measuring the knowledge, skills, and confidence for self-management. Individuals respond to items such as ‘I know how to prevent further problems with my health condition’ using a 4-point Likert scale ranging from ‘strongly disagree’ (1) to ‘strongly agree’ (4). A ‘not applicable’ (N/A) response is also available. Responses of N/A are scored as 0 and are reported to distinguish those left blank. A continuous activation score is computed from the raw score using an empirically derived calibration table by Insignia Health. The PAM-13 is scored along a Guttman scale (0–100) with higher scores along a unidimensional continuum signifying greater activation. Level 1 (PAM-13 score ≤47; disengagement and disbelief about one’s own role in self-management) encompasses items 1 and 2; Level 2 (47.1–55.1; increasing awareness, confidence, and knowledge in self-management tasks), items 3–8; Level 3 (55.2–67; readiness and taking action), 9–11; and Level 4 (≥67.1; sustainment).

## Box 1. A perspective of a non-dialysis patient on the role of patient activation and self-management.

As a patient being told that I had some loss of kidney function (kidney disease) my own knowledge, skills, ability, and confidence to look after my own kidney health were not immediately activated in any way. The fact that my kidneys were becoming less effective seemed to be related to the normal aging process as I am in my early 70’s. The only direct action by my GP was to point me in the direction of the Kidney Lifestyle Team project at University Hospitals Leicester and to ask if I would like to assist in their patient advisory group work. Even then with only yearly blood tests and no specific GP guidance on the results regarding the slow decline in my kidney function, the lack of any outward signs or symptoms of my deteriorating body kidney functions; I just parked this whilst noting that diet and exercise were probably something that I could do to help my condition. Being overweight compared with norm values is something that I have been for most of my life; I try to eat fairly healthily, although I do tend to raid the chocolate and biscuits especially in the winter months when there are less apples on the trees to snack on. Exercising routinely is not something that I have at any time in my life been inspired to take up, so walking is my general limit. During my life I have spent many hours sat at a desk, workbench, or computer to carry out my work and into semi-retirement I continue with such ways of working with the addition of regular video conference calls.

Until recently, I was still not bringing together my own Patient Activation factors to do something really positive and to accept that I have kidney disease. In taking the PAM test I suddenly began to appreciate that with the statements from 8) through to 13) I was actually circling the ‘disagree with’ as a reflection of my current state. As a fairly intelligent person, this was of great concern to me and here again Lifestyle changes were placed securely on my doorstep and something that I need to address along with the other statements that I could not show a positive answer to. But being a normal human being, without any activators, targets or plans my actual motivation to do something is again drifting in to further neglect.

72 year old male living with non-dialysis dependent kidney disease

## Box 2. Perspective of a dialysis patient on the role of patient activation and self-management.

When I started dialysis in 1998 as a 14 year old, the actions, behaviour and ethos of the clinical team in paediatrics were entirely focused on empowering and supporting me to self-manage and take complete control of my dialysis care. Life had to come first, so home dialysis was a no brainer. I had never heard the term Patient Activation, which as it happens, is not one I particularly like since to me it implies that patients are passive, disengaged beings just waiting for somebody to come along and “activate” them. But I absolutely support the concept and recognise the need to define and measure it, plus fussing over nomenclature can be distracting from the main point. Reflecting back over my now 23 years of hugely successful, stable and incredibly uneventful solo home dialysis (5 years PD then 18 years nocturnal HD so far), plus armed with the knowledge I now have from many years of patient advocacy, peer supporting and renal QI work, I have observed that the set of ingredients I brought to the table when I was first diagnosed played an enormous part in my ability to learn and reduce my dialysis to a mere footnote in my day to day busy life. I came from a tight supportive family; my dad was a doctor and I was at a school that bent over backwards to support my education whilst I spent months in hospital. My friends stuck by me and my parents had a wide support network to care for my brothers so they could focus on my needs at crisis points. We also, critically, were financially secure. I was hyper conscientious and academic, so despite a catastrophic run of serious health complications and lengthy hospitalisations in the early years, I was still able to get top GCSE and A-Level grades to secure my place at Cambridge University, all of which were invaluable to my future prospects and career. In short, my privilege, educational and socio-economic circumstances had by far the biggest influence over the fact that I was extremely “activated” from the start. Subsequently, my knowledge, confidence, and an equal, respectful partnership with my healthcare team, as well as ability to nimbly navigate the behemoth that is the NHS and to fight in my corner when needed, has saved me from many complications by enabling me to rapidly secure the interventions I need, or to just get my many repeating care tasks and dialysis “admin” done with minimal disruption to my working days. My personal circumstances mean that I am mentally able to just keep ploughing on broadly unaffected by aspects of kidney failure which can entirely destroy some other people’s lives and livelihoods.

As such, I feel that in the dialogue around Patient Activation it is critical that we give due consideration to people’s external lives and the inequalities which can affect their capacity and capability to take on self-management of a challenging health condition through no fault of their own.

38 year old female living on home haemodialysis
